# Inhibition of Spinal Oxidative Stress by Bergamot Polyphenolic Fraction Attenuates the Development of Morphine Induced Tolerance and Hyperalgesia in Mice

**DOI:** 10.1371/journal.pone.0156039

**Published:** 2016-05-26

**Authors:** Filomena Lauro, Luigino Antonio Giancotti, Sara Ilari, Concetta Dagostino, Micaela Gliozzi, Chiara Morabito, Valentina Malafoglia, William Raffaeli, Maurizio Muraca, Bianca M. Goffredo, Vincenzo Mollace, Carolina Muscoli

**Affiliations:** 1 San Raffaele Roma S.r.l., Roccelletta di Borgia, Catanzaro, Italy; 2 Department of Surgical Sciences, Parma University, Parma, Italy; 3 YAP (Young Against Pain) Collaboration, Parma, Italy; 4 Institute of Research for Food Safety & Health (IRC_FSH), Department of Health Sciences, University “Magna Graecia” of Catanzaro, Catanzaro, Italy; 5 Department of Experimental Medicine, “Sapienza” University of Rome, Rome, Italy; 6 Institute for Research on Pain, ISAL-Foundation, Torre Pedrera (RN), Italy; 7 Research Laboratories, Children’s Hospital “Bambino Gesù” Research Institute, Rome, Italy; University of Texas Medical Branch, UNITED STATES

## Abstract

*Citrus Bergamia Risso*, commonly known as Bergamot, is a fruit whose Essential Oil and Bergamot Polyphenolic Fraction have numerous medicinal properties. It is also an excellent antioxidant and in this study, for the first time, its potential effect on morphine induced tolerance in mice has been investigated. Our studies revealed that development of antinociceptive tolerance to repeated doses of morphine in mice is consistently associated with increased formation of superoxide, malondialdehyde and tyrosine-nitrated proteins in the dorsal horn of the spinal cord such as the enzyme glutamine synthase. Nitration of this protein is intimately linked to inactivation of its biological function and resulting increase of glutamate levels in the spinal cord. Administration of Bergamot Polyphenolic Fraction (5–50 mg/kg) attenuated tolerance development. This effect was accompanied by reduction of superoxide and malondialdehyde production, prevention of GS nitration, re-establishment of its activity and of glutamate levels. Our studies confirmed the main role of free radicals during the cascade of events induced by prolonged morphine treatment and the co-administration of natural derivatives antioxidant such as Bergamot Polyphenolic Fraction can be an important therapeutic approach to restore opioids analgesic efficacy.

## Introduction

For centuries, opioid drugs have been the mainstay of pain treatment [1], but long-term morphine administration leads to analgesic tolerance [2]. Tolerance to the analgesic effects of opiates results from the complex changes in various molecular and biochemical pathways [3]. Numerous data have demonstrated the involvement of superoxide (SO, O_2_^**•-**^) and peroxynitrite (PN, ONOO^-^) (the product of the diffusion-controlled reaction of SO with nitric oxide, NO) in the development of chronic pain, in the transition of acute to chronic pain as well as in the opiate-induced hyperalgesia and antinociceptive tolerance. Peroxynitrite is considered the major agent responsible for tyrosine-nitration proteins in the dorsal horn of the spinal cord, including the mitochondrial isoform of superoxide dismutase (MnSOD), the glutamate transporter GLT-1, the glutamate receptor NMDA (N-methyl-D-aspartate) and the enzyme glutamine synthase (GS) [4, 5]. Furthermore, antinociceptive tolerance and PN production have been associated with activation of spinal cord glial cells, as well as production of pro-inflammatory cytokines such as TNF-α, IL-1β and IL-6 and spinal sensitization and activation of the nuclear enzyme poly(ADP-ribose) polymerase (PARP) [4, 6].

Bergamot *(Citrus bergamia*, *Rissu)* is an endemic plant of the Calabrian region in Southern Italy with a unique profile of flavonoid and flavonoid glycosides present in its juice and albedo, such as neoeriocitrin, neohesperidin, naringin, rutin, neodesmin, rhoifolin and poncirin. Bergamot differs from other citrus fruits not only because of the composition of its flavonoids, but also because of their particularly high concentration [7]. It is used in cosmetics and food, but it has also numerous medicinal properties: bergamot peel is a potential source of natural antimicrobials that are active against Gram-negative bacteria [8], and in vapour form has been shown to have antimicrobial effects against both bacteria and fungi [9]; Bergamot Essential Oil (BEO) has also a neuroprotective function, in fact it reduces neuronal damage caused *in vitro* by excitotoxic stimuli and this neuroprotective effect was associated with prevention of injury-induced engagement of critical death pathways [10]. Bergamot polyphenols have hypolipemic and hypoglycaemic activity: given orally for 30 days to both rats and patients reduces total cholesterol, LDL (an effect also accompanied by elevation of cHDL) and triglyceride levels [7]. Furthermore, in a model of carotid arteries injured by balloon angioplasty, balloon injury led to a significant restenosis with smooth muscle cell proliferation and neointima formation, accompanied by increased expression of LOX-1 receptor, malondialdehyde, superoxide formation and nitrotyrosine staining. Pretreatment of rats with BEO-NVF (nonvolatile fraction) reduced the neointima proliferation, LOX-1 expression, MDA overproduction, nitrotyrosine staining and *in situ* ROS generation in a dose-dependent manner [11].

This data evidenced as bergamot extract is an excellent antioxidant, in fact it exhibited 100% antioxidant activity in the aldehyde/carboxylic acid assay [12]. In particular, bergamot polyphenols are contained mainly in the albedo of the bergamot fruit, thereby getting high concentrations in bergamot juice after the fruit is squeezed [7]. In this study, we evaluated the ability of BPF (Bergamot Polyphenolic Fraction) to inhibit tolerance induced by morphine-repeated administration. Our data showed for the first time, that co-administration of morphine and BPF can be a good therapeutic approach for the treatment of chronic pain thanks to its ability to inhibit, in a dose-dependent manner, the development of tolerance preserving analgesic-morphine property.

## Materials and Methods

### Animals

Male CD-1 mice (24–30 g; Charles River Laboratory) were housed and cared for in accordance with the guidelines of the University of Tor Vergata, Rome, Italy, as well as complied with the Italian regulations for the protection of animals used for experimental and other scientific purposes, and with European Economic Community regulations (2010/63/UE). The study was approved by Italian Ministry of Health (permit number 144/2012-B). Mice were housed 5–7 per cage and maintained under identical conditions of temperature (21 ± 1°C) and humidity (60% ± 5%) with a 12-hour light/12-hour dark cycle and allowed food *ad libitum*. Natural antioxidant BPF was kindly provided by H&AD (Herbal and Antioxidants Derivatives srl). All others drugs were purchased from Sigma and dissolved in saline (sodium chloride 0,9%). The following experimental groups were used:

#### Acute morphine groups

Mice received a single s.c. injection of morphine (1, 3, 6 mg/kg, n = 15 each dose). Behavioral tests have been carried out after 30, 45, 60 minutes from the treatment.

#### Naive group

In this group, mice (n = 15) were injected twice a day with an i.p. injection of saline (vehicle used to deliver the drugs to the other groups over 4 days) and a s.c. injection of saline (vehicle used to deliver morphine to the other groups over 4 days). On day 5 mice received an i.p. injection of saline followed 15 minutes later by a s.c. injection of morphine (3 mg/kg).

#### BPF group

In this group, mice (n = 15) were injected twice a day for 5 days with an i.p. injection of the highest dose of BPF (50 mg/kg) followed by s.c. injection of saline.

#### Morphine group

In this group, mice (n = 15) were injected twice a day for 4 days with an i.p. injection of saline and a s.c. injection of morphine (20 mg/kg). On day 5 mice received an i.p. injection of saline followed 15 minutes later by a s.c. dose of acute morphine (3 mg/kg).

#### Morphine plus drug groups

In this group, mice (n = 15) were injected twice a day for 4 days with an i.p. injection of different doses of BPF (5, 25 and 50 mg/kg), L-NAME (10 mg/kg) and MnTBAP (10 mg/kg) followed by s.c. injection of morphine (20 mg/kg). On day 5 mice received an i.p. dose of drugs, followed 15 minutes later by the s.c. dose of acute morphine (3 mg/kg).

All doses were given in a 0.1 ml volume at approximately 7 am and 4 pm.

On day 5 and after the behavioral tests, carried out after 60 minutes from the treatment, the animals were euthanized via decapitation with a guillotine assuring that discomfort and pain to animals was limited to that which was unavoidable for the conduct of scientifically valuable research. Spinal cord tissues from the lumbar enlargement segment of the spinal cord (L4–L5) were removed and tissues stored at -80°C for subsequent analysis.

### Hot plate test

Nociceptive thresholds were determined by measuring latencies (in seconds) of mice placed in a transparent glass cylinder on a hot plate (Panlab) maintained at 52°C. Determination of antinociception was assessed between 7:00 am and 10:00 am. Responses indicative of nociception included intermittent lifting and/or licking of the hindpaws or escape behavior. Hot plate latencies were taken in mice from all groups on day 5 before (baseline latency) and 50 minutes after an acute dose of morphine (3 mg/kg). A cut-off latency of 25 seconds was employed to prevent tissue damage. 15 mice per group were used, and all experiments were conducted with the experimenters blinded to treatment conditions. Results are expressed as percentage of maximum possible antinociceptive effect, which was calculated as follows: (response latency − baseline latency) / (cut-off latency − baseline latency) × 100.

### *In situ* detection of superoxide anion

Detection of superoxide anion was evaluated by hydroethidine (HE) administration. 50 min after challenge dose of morphine administration mice were treated with 200μl of PBS containing 1μg/μl of hydroethidine (Molecular Probes) and 1% dimethylsulfoxide (DMSO). Spinal cords were removed 15 min later and frozen in nitrogen. Sections of 5 μm were cut by a cryostat and examined by fluorescence microscope (excitation 510nm, emission 580) for the evaluation of ethidium bromide accumulation, the oxidation product of hydroethidine.

### Immunoprecipitation and Western blot analyses

Dorsal half of the spinal cord lumbar region enlargement (L4–L5) were obtained as described previously [13, 14, 15]. The resulting lysates samples were stored immediately at –80°C and immunoprecipitation of tyrosine nitrated protein followed by Western blot analysis performed as previously described [13, 14]. For immunoprecipitation of nitrated proteins, a well-characterized affinity-purified anti-nitrotyrosine monoclonal antibody conjugated to agarose beads from Upstate Biotechnology was used according to the manufacturer’s instructions. To determine whether GS was nitrated, Western blot analysis of i.p. protein complex and total lysates were performed using antibodies specific to these proteins. Briefly, the immunoprecipitated proteins were resolved in 10% SDS-PAGE mini and proteins transferred to nitrocellulose membranes. Membranes were blocked for 2 hours at room temperature with 5% BSA in TBST 0.1%, followed by overnight incubation with monoclonal antibody for GS (1:1000; Cayman Chemical). Membranes were then washed with TBS/T and incubated secondary antibodies conjugated to peroxidase for 1 hour at room temperature. After washes, protein bands were visualized by enhanced chemiluminescence (Amersham Biosciences). After stripping, total lysates membranes were reprobed with either monoclonal anti β-actin antibody (1:2000; Sigma-Aldrich) as a loading control. The relative expression of the protein levels as the band density for GS (45 kDa) and β-actin (50 kDa) were quantified by scanning of the X-ray films with MSF-300G and a computer program.

### Malonylaldehyde detection

MDA quantification was performed through thiobarbituric acid reactive substances (TBARS) assay. In brief, tissue sample were added to a vial containing 10% NaOH, 20% Acetic Acid and TBA. The vials were boiled at 90–100°C and after 1h the tubes were placed in ice to stop reaction. Before being transferred to a black 96-well microtiter plate samples were centrifugated 10 minutes at 1600 x g at 4°C. The MDA-TBA adduct was measured fluorometrically at an excitation wavelength of 530 nm and emission wavelength of 550 nm using Infinite 200 microplate fluorometer (Tecan).

### Analysis of spinal cord amino acids

Dorsal half of the spinal cord lumbar region enlargement (L4–L5) were washed with 0,15 M NaCl, weighed and homogenized at 4°C with cold PBS. Subsequently was added 5% salicylic acid and kept at RT for 15 minutes. Homogenated was centrifuged at 11.000 g for 15 minutes at 4°C and supernatant was stored at 20°C for the subsequent HPLC analysis.

### Statistical analysis

Results are given as mean ± SEM. Statistical analysis was performed using ANOVA followed by Student- Newman-Keuls. P<0.05 was considered statistically significant.

## Results

### The development of morphine-induced tolerance is inhibited by antioxidant

**Fig 1:**
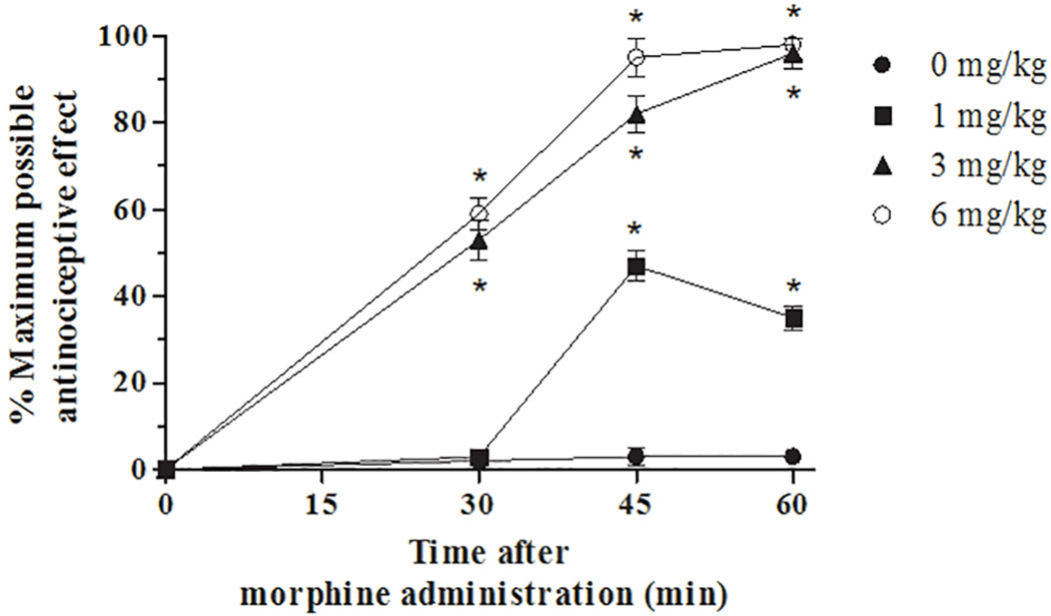
Morphine dose-response curve analysis. Acute injection of morphine (3 mg/kg), in mice, produced a significant near-maximal antinociceptive response until 60 minutes. Results are expressed as mean ± SEM for 15 mice;*P<0.001 vs morphine 0 mg/kg.

**Fig 2:**
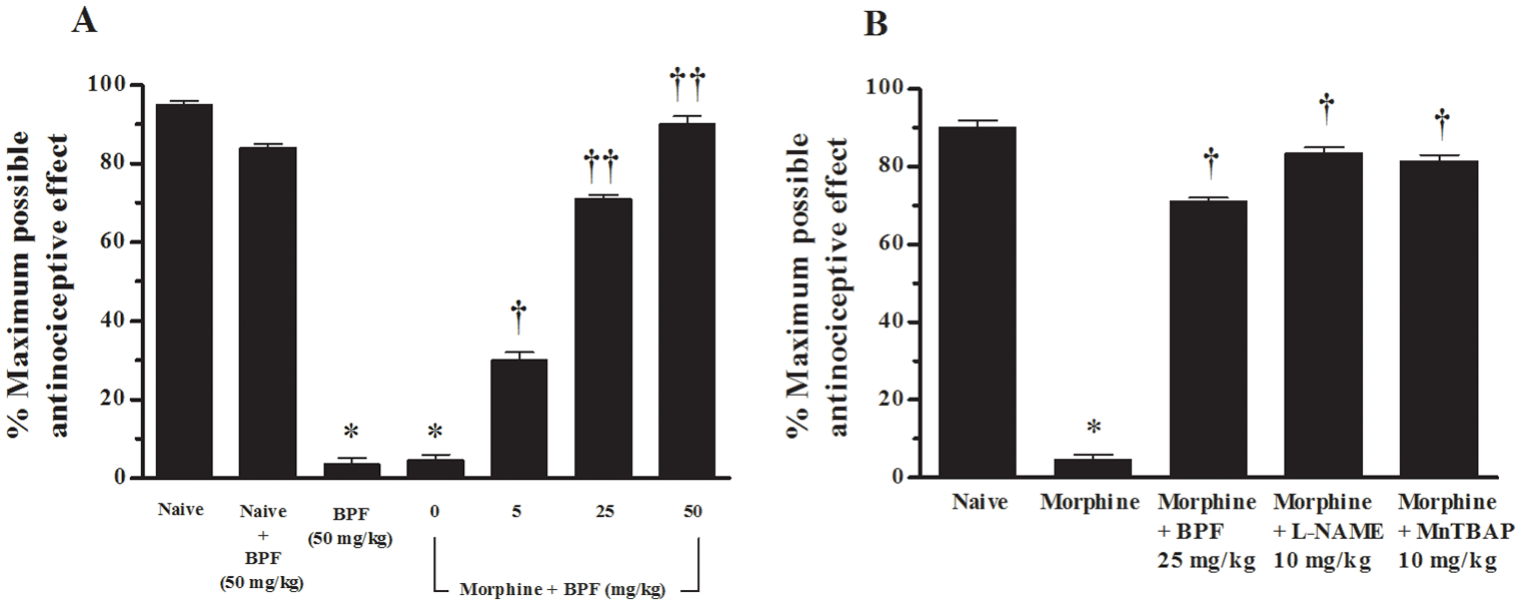
The development of morphine antinociceptive tolerance is prevented by substances with antioxidant properties. (A-B) A significant loss to the antinociceptive effect of the acute injection of morphine was observed in animals that received repeated administration of morphine over 4 days. (A) Co-administration of morphine over 4 days with BPF (5–50 mg/kg) inhibited the development of tolerance in a dose-dependent manner. When tested alone, the highest dose of BPF (50 mg/kg) did not have antinociceptive effects. (B) In morphine-treated mice, the effect of BPF (25 mg/kg) was comparable to L-NAME (10 mg/kg) or MnTBAP (10 mg/kg). Results are expressed as mean ± SEM for 15 mice. *P<0.001 vs Naive; †P<0.01 vs morphine; ††P<0.001 vs morphine.

Acute injection of morphine (3 mg/kg), in animal that received saline over 4 days (vehicle group), produced a significant near-maximal antinociceptive response until 60 minutes after administration (Figs 1 and 2) compared with animals receiving an equivalent injection of saline (naive group).

**Fig 3:**
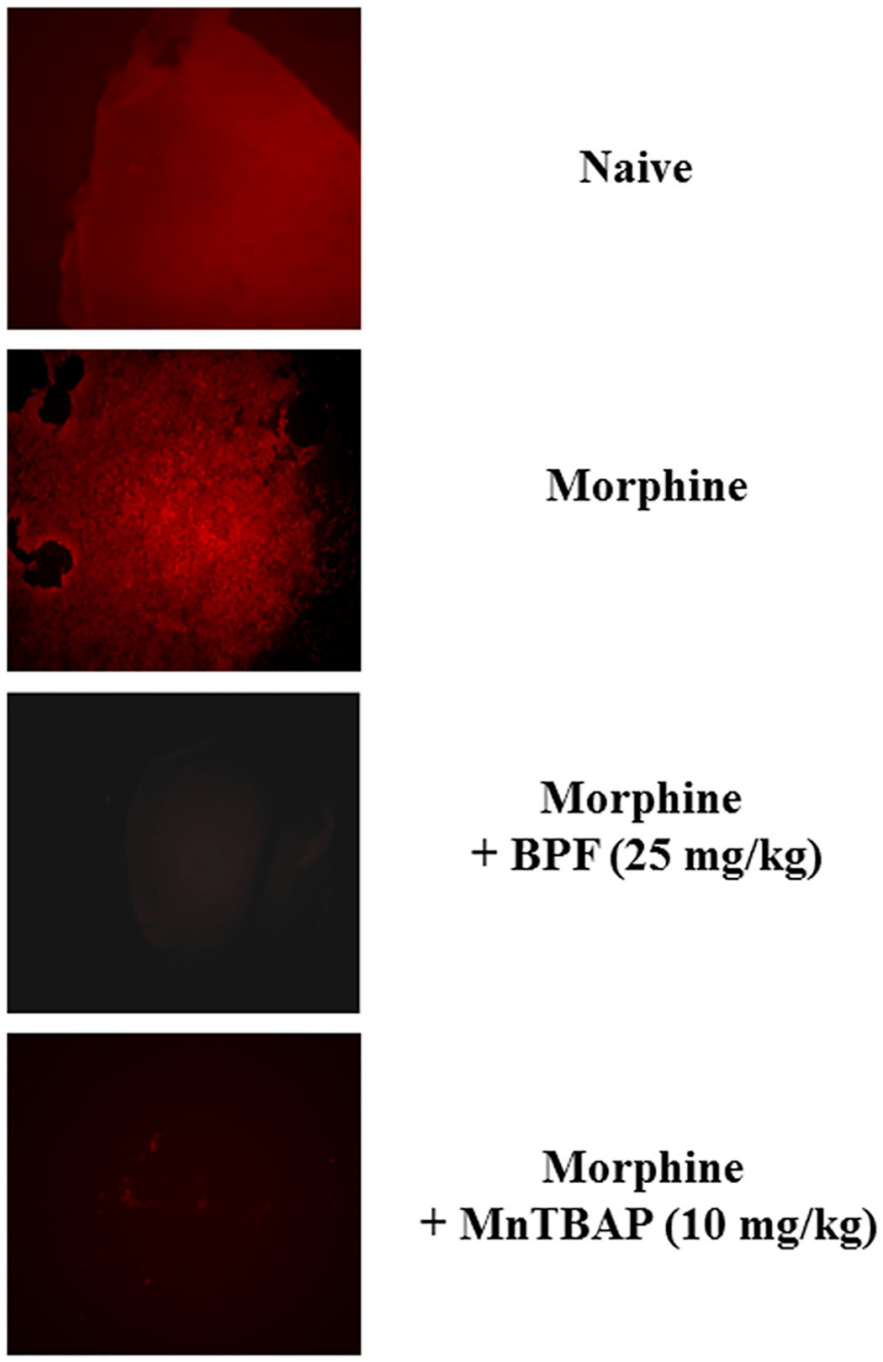
Chronic morphine administration is associated with increased superoxide formation in the L4-L5 portion of spinal cord. Chronic morphine administration induced an overproduction of O_2_^·-^ in the spinal cord compared with mice naïve group as demonstrated by HE oxidation. BPF (25 mg/kg; n = 15) or MnTBAP (10 mg/kg) co-administration was able to reduce O_2_^·-^ chronic morphine-induced increase. Original magnification, ×10. Micrographs are representative of at least 3 from different animals in experiments performed on different days.

Repeated morphine administrations over the same time course (morphine group) led to the development of antinociceptive tolerance, as evidenced by a significant loss of its antinociceptive response (Fig 2). Tolerance is associated to superoxide production in the L4-L5 portion of the spinal cord (Fig 3). To investigate whether the increased superoxide synthesis had a functional role in the development of morphine antinociceptive tolerance, morphine was co-administered with antioxidants such as BPF (Table 1) [16]. Co-administration of morphine with BPF inhibited in a dose-dependent manner (5–50 mg/kg/d, n = 15) the development of antinociceptive tolerance (Fig 2). To confirm the important antioxidant effect of BPF in the morphine tolerance, we compared BPF action with L-NAME, a nonselective NOS inhibitor N-nitro-L-arginine methyl ester, and MnTBAP, the nonselective O_2_^−^ scavenging agent [4]. The effect of L-NAME or MnTBAP was similar to BPF and no significant difference have been observed in the capacity of inhibiting the development of morphine tolerance antinociceptive (Fig 2B). In naïve mice, the highest dose of BPF (50 mg/kg) not influenced the response to acute morphine dose (3 mg/kg) excluding an interaction between morphine and antioxidants. When tested alone at the highest dose, L-NAME (10 mg/kg) and MnTBAP (10 mg/kg) [4, 17] BPF (Fig 2A), did not have antinociceptive effects evaluated with hot plate test (range 6/7 seconds).

**Table 1:** Bergamot polyphenolic fraction components.

Apigenin-6-8-di-C-glucoside
Diosmetin-6-8-di-C-glucside
Luteolin-6-8-di-C-glucoside
Diosmetin-8-C-glucoside
Diosmetin-7-O-glucoside
4H-1-Benzopyran-4-one, 7-dihydroxy-2-(4-hydroxy-3-methoxyphenyl)
Luteolin-7-O-neohesperidoside
Rhoifolin
Neodiosmin
Diosmetin-7-O-neohesperidoside-6′′-O-HMG
Apigenin-7-O-neohesperidoside-6′′-O-HMG
Esperetin-7-O-glucoside
Neoesperidin
Naringin
Neoeriocitrin
Eriodictyol-7-O-neohesperidoside-6′′-O-HMG
Bruteridin
Melitidin
Hesperetin-7-O-glucoside-6′′-O-HMG
Naringenin-7-O-glucoside-6′′-O-MHG

### BPF inhibits superoxide and MDA increase in morphine-tolerant mice

**Fig 4:**
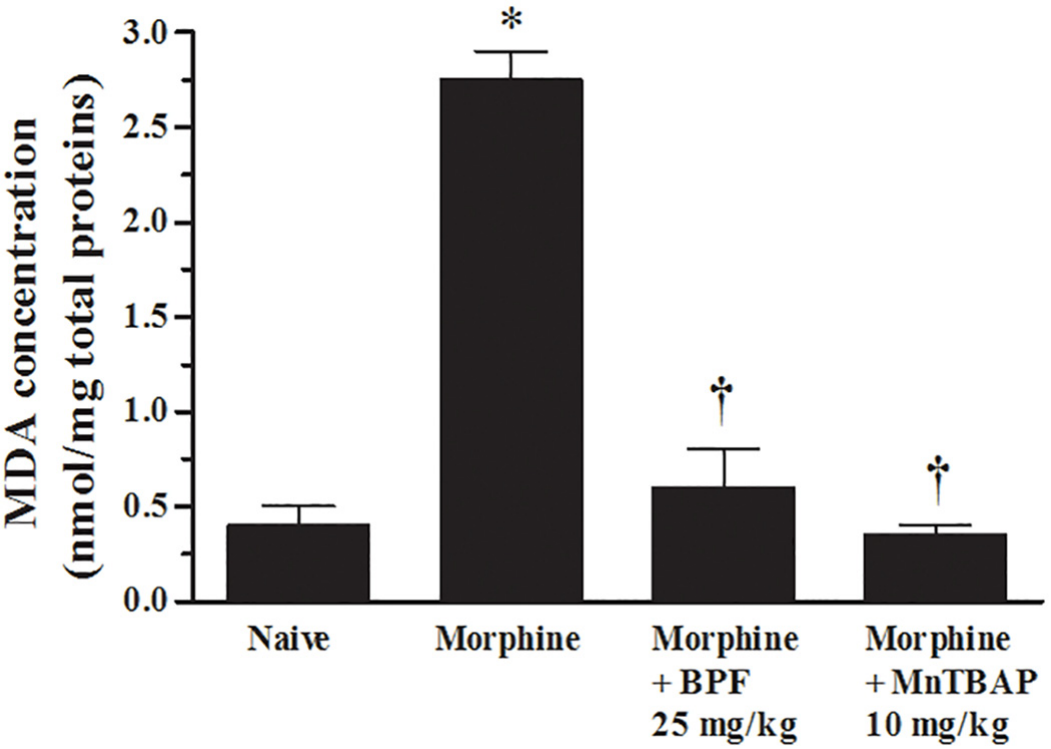
MDA assay demonstrated increased lipid peroxidation in tolerant mice. Spinal cord extract from morphine group demonstrated a significant increase in MDA that was reduced by BPF (25 mg/kg) as well as by MnTBAP (10 mg/kg). Results are expressed as mean ± SEM for 15 rats. *P<0.001 vs Naive; †P<0.01 vs morphine.

Hydroethidine (HE), when oxidated by superoxide, forms the fluorescent cation ethidium [18, 19], the presence of the fluorescent probe evidenced superoxide production. Our data demonstrated that chronic morphine administration induced an increase of superoxide production as showed by the presence of ethidium (Fig 3). Furthermore, to confirm the formation of radical species during morphine tolerance, we quantified a well-known oxidative stress marker, the malondialdehyde (MDA), a highly reactive three carbon dialdehyde produced as a byproduct of polyunsaturated fatty acid peroxidation and arachidonic acid metabolism. Prolonged morphine treatment induced an increase of MDA level (Fig 4) evaluated through MDA-TBA assay. Co-administration of morphine with BPF or MnTPAB for over 4 days inhibited antinociceptive tolerance to morphine (Fig 2) together with superoxide formation (Fig 3) and MDA levels (Fig 4). Therefore, acute morphine administration, in naïve animals, had not showed a significant ethidium production (Fig 3).

### The development of morphine-induced tolerance is associated with tyrosine nitration and this is inhibited by BPF

**Fig 5:**
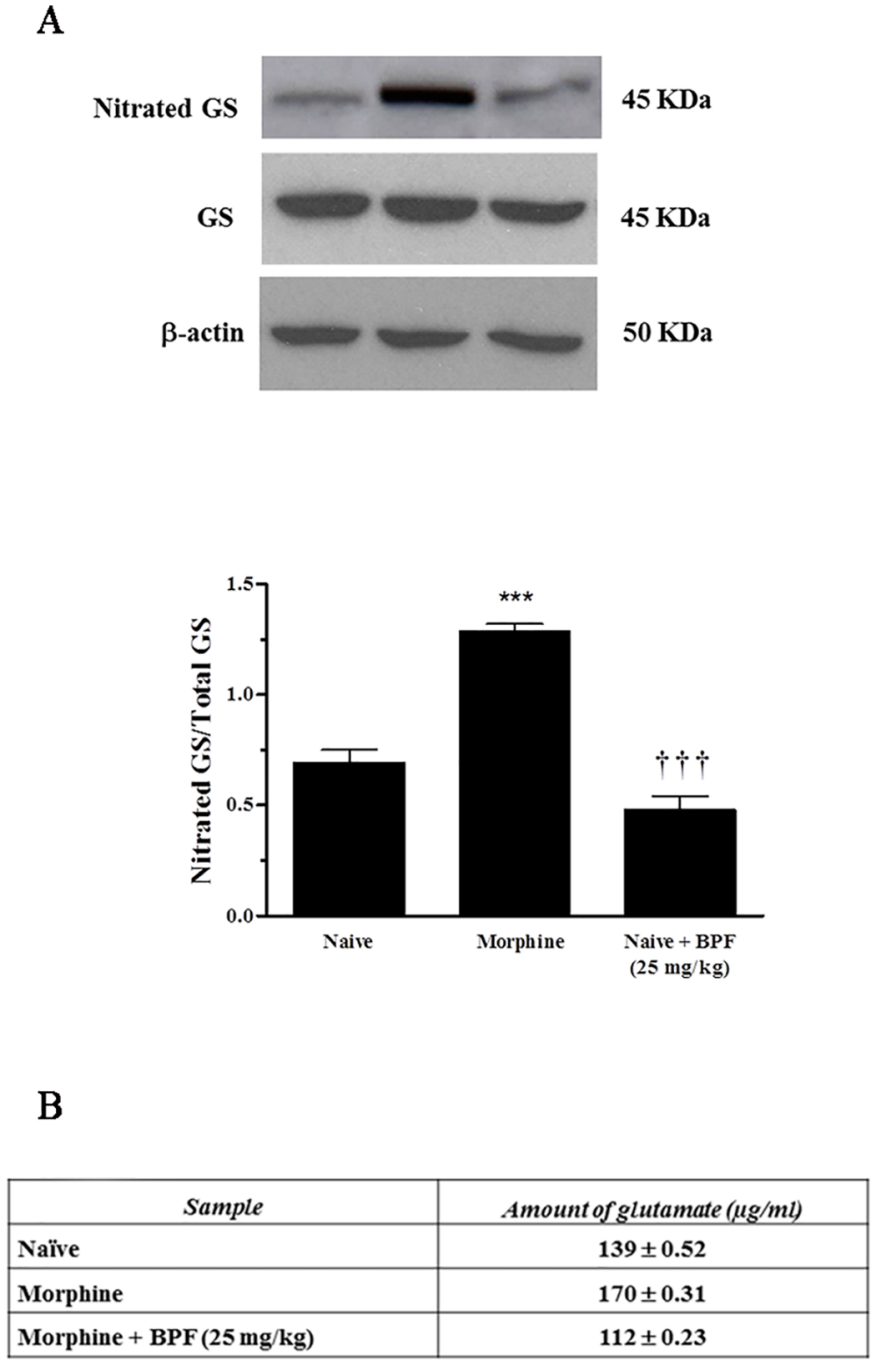
Morphine antinociceptive tolerance is associated with nitroxidative stress which is blocked by BPF. (A) When compared with naive mice, morphine group lead to significant nitration of GS in the L4-L5 portion of the spinal cord tissues as measured by immunoprecipitation. Co-administration of morphine over 4 days with BPF (25 mg/kg) prevented GS nitration. Total protein levels did not change among groups as measured by Western blotting analysis. (B) Posttranslational nitration of GS led to functional enzymatic inactivation as evidenced by increased amount of glutamate. Co-administration of morphine with BPF (25 mg/kg) restored the enzymatic activity of GS. Gels shown are representative of gel results obtained from six animals and of at least three experiments performed on different days. ***P<0.001 vs Naive; †††P<0.001 vs morphine.

The development of morphine tolerance was associated with an increase of tyrosine-nitrated proteins in the L4-L5 portion of the dorsal horn such as the spinal glutamine synthetase (GS) (3±0.65 densitometric units for naïve group, 183.67±0.51 for morphine group; n = 5, P<0.001), (Fig 5A) an astrocytic enzyme that converts glutamate to its nontoxic analogue, glutamine [20]. The inhibition of antinociceptive tolerance by BPF was associated with an attenuation of posttranslational nitration of GS in the spinal cord (183.67±0.51 densitometric units for morphine group, 10.34±0.93 for morphine plus BPF; n = 5, P<0.001) (Fig 5A). Nitration of these proteins is intimately linked to inactivation of their biological function. Indeed, amino acid HPLC analisys, showed an increase of glutamate levels in morphine treated mice (Fig 5B), while BPF co-administration is able to restore basal glutamate level (Fig 5B). GS inactivation has important ramifications manifested by enhancing glutamatergic neurotransmission, which is key to central sensitization [21]. Therefore, our studies demonstrated that natural compounds with antioxidant activity are able to restore the analgesic efficacy of morphine via inhibition of free radicals agents, that are crucially for the cascade events induced by prolonged morphine administration.

## Discussion

Opioid narcotics, such as the mainstay morphine, are the most effective treatments for acute and chronic severe pain but their clinical utility is nearly always hampered by the development of analgesic tolerance as well as painful hypersensitivity now known as morphine-induced hyperalgesia. Typically, the development of tolerance to morphine treatment necessitates escalating doses to achieve equivalent pain relief [22]. The underlying mechanisms are still poorly understood but several data associate nitroxidative stress to opioids tolerance and hyperalgesia [4, 23]. In particular, substantial evidence accumulated over the last years suggests that NMDA receptors and NO play an important role in the development of morphine tolerance [4, 5, 24]. Following repeated opioid administrations excessive excitation of the NMDA occurs indirectly via the activation of μ-opioid receptors. Activation of μ-receptor results, in turns, in indirect NMDA receptor activation by initiating a second-messenger PKC translocation to the membrane. In fact, PKC translocation activates the NMDA receptor by inducing the removal of Mg^2+^ blockade with consequent increased intracellular Ca^2+^ influx.

Increased _i_Ca^2+^ leads to both activation of NO synthase followed by subsequent release of nitric oxide [25] and production of superoxide from mitochondria [26].

Simultaneous generation of these two molecules, SO and NO, favors the production of peroxynitrite, a very potent initiator of DNA strand breakage, which, in turn, initiates the production of the nuclear repair enzyme PARP. It was demonstrated that removal of SO and in turn of peroxynitrite inhibits poly-ADP-ribose-polymerase activation together with the inhibition of morphine tolerance [4, 14, 27].

In the present study, we decided to test for the first time, the efficacy of Bergamot Polyphenolic Portion (BPF) in this phenomena.

We observed that repeated administration of morphine for several consecutive days produced tolerance to the opioid and an increase in superoxide formation in the L4-L5 portion of the mice spinal cord. BPF co-administration was able to inhibit morphine antinociceptive tolerance reducing O_2_^**·**-^ chronic morphine-induced increase.

Epidemiological studies have shown that food has a direct impact on the health. In fact, plant derived foods such wine, fruits, nuts, vegetables, grains, legumes exert some beneficial effects on diseases [28]. The capacity of some plants to reduce the risk of chronic diseases is due to the presence of non-nutrient secondary metabolites, known phytochemicals, that exerts a wide range of biological activities. These metabolites include various groups of polyphenols (anthocyanins, flavones, isoflavones) and terpenoids (carotenoids, monoterpenes, and phytosterols). Their bioactivity has been associated to their antioxidant properties that are involved in the onset development of many of the chronic degenerative diseases such as cancer and inflammation [29, 30].

Recently, some studies have investigated the effect of some fruit’s and herb’s extracts as resveratrol, quercetin, curcumin, lycopene, myricitrin, genistein, and (-)-linalool on differents experimental animal models of chronic pain [31–37].

An our recent study show that in a rodent model of opiate tolerance, removal of the free radicals with phenolic compounds of olive oil such as hydroxytyrosol and oleuropein re-instates the analgesic action of morphine. Chronic injection of morphine in mice led to the development of tolerance and this was associated with increased nitrotyrosine and MDA formation together with nitration and deactivation of MnSOD in the spinal cord. Removal of free radicals by hydroxytyrosol and oleuropein blocked morphine tolerance by inhibiting nitration and MDA formation and replacing the MnSOD activity. Thus, the analgesic effect *in vivo* of phenolic fraction of virgin olive oil derives from its antioxidant activities [38].

An excess of major pain neurotransmitter glutamate contributes to morphine hyperalgesia, in fact, stimulating NMDA receptors favors the maintenance of hyperalgesia via SO [5, 13] and NO formation [39, 40, 41, 25].

Removal of glutamate reduces the excitatory signals and prevents the persistence of excitotoxic levels of glutamate in the synaptic cleft. In the glial cells, glutamate is transformed in the non-toxic glutamine by the enzyme glutamine synthetase. Glutamine synthetase can be, in turn, inactivated by oxidation of one of the 16 histidine residues in the glutamine synthetase catalytic site [42] and nitration of GS tyrosine residues was also associated to morphine tolerance [4].

We observed that glutamate level seems to be associated to GS nitration. Furthermore, treatment of morphine-tolerant mice with BPF resulted in a reduction of SO production, MDA formation, prevention of GS nitration and re-establishment of glutamate levels leading to tolerance inhibition. These data highlight the importance of free radicals removal for the maintenance of the correct therapeutic function of opioid during pain state.

The use of natural antioxidant for the treatment of pain condition diseases, can be considered a new valid approach, effective and affordable, useful to control the disease development and progression. Natural products could be good remedy as they are better tolerated due to the less showed side effects.

Therefore, our translational research can constitute a new therapeutic approach for patients with chronic pain treatment and can help to improve their quality of life.
